# Aqueous Ammonia Pre-treatment of Wheat Straw: Process Optimization and Broad Spectrum Dye Adsorption on Nitrogen-Containing Lignin

**DOI:** 10.3389/fchem.2019.00545

**Published:** 2019-08-02

**Authors:** Mika Henrikki Sipponen, Monika Österberg

**Affiliations:** Department of Bioproducts and Biosystems, School of Chemical Engineering, Aalto University, Espoo, Finland

**Keywords:** biorefinery, environment, lignocellulose, plant biomass, sustainable materials, water purification

## Abstract

Biorefineries need cost-efficient pretreatment processes that overcome the recalcitrance of plant biomass, while providing feasible valorization routes for lignin. Here we assessed aqueous ammonia for the separation of lignin from hydrothermally pretreated wheat straw prior to enzymatic saccharification. A combined severity parameter was used to determine the effects of ammonia concentration, treatment time and temperature on compositional and physicochemical changes [utilizing elemental analysis, cationic dye adsorption, FTIR spectroscopy, size-exclusion chromatography (SEC), and ^31^P nuclear magnetic resonance (NMR) spectroscopy] as well as enzymatic hydrolysability of straw. Pretreatment at the highest severity (20% NH_3_, 160°C) led to the maximum hydrolysability of 71% in a 24 h reaction time at an enzyme dosage of 15 FPU/g of pretreated straw. In contrast, hydrolysabilities remained low regardless of the severity when a low cellulase dosage was used, indicating competitive adsorption of cellulases on nitrogen-containing lignin. In turn, our results showed efficient adsorption of cationic, anionic and uncharged organic dyes on nitrogen-containing lignin, which opens new opportunities in practical water remediation applications.

## Introduction

The hierarchical and recalcitrant structure of renewable plant biomass hampers its enzymatic hydrolysis for the production of biofuels (Himmel et al., 2007; Chundawat et al., 2011a,b). One of the main constraints arises from the presence of lignin that functions as a natural resin within and between plant cell walls, and adsorbs cellulases in the saccharification step (Chen and Dixon, 2007; Li et al., 2016; Liu et al., 2016; Sipponen et al., 2017a). Various acid, base, and solvent-based pretreatments increase saccharification yields by removing/altering lignin and by causing complex physical-chemical changes in the plant cell walls (Paës et al., 2017).

Hydrothermal and thermochemical pre-treatments have predominated in commercial and pre-commercial biorefinery concepts (Pihlajaniemi et al., 2016; Auxenfans et al., 2017). Although steam-explosion, autohydrolysis, and dilute acid hydrolysis pre-treatments require no or low chemical input, these processes do not generate a soluble lignin fraction. Instead, lignin ends up to the residual solids after incomplete enzymatic hydrolysis (and fermentation) of the pre-treated biomass. Such residual lignin is usually heavily contaminated with unhydrolyzed cellulose. On the contrary, organosolv and alkaline pre-treatments dissolve lignin and allow for its isolation in a relatively pure form (Hage et al., 2009; Mousavioun and Doherty, 2010). This is important since besides its detrimental effects on enzyme activity, lignin is a potential raw material for aromatic chemicals (Zakzeski et al., 2010) and biobased polymers (Laurichesse and Avérous, 2014), with substantial effect on the profitability of cellulosic ethanol plants (Ragauskas et al., 2014).

Lignin is thus indirectly and directly involved in the two central challenges of the 2G bioethanol production: (1) Development of cost-efficient pre-treatments to open up the recalcitrant structure of lignocellulose (2) Demonstration of applications for lignin that secure sufficient volume and profits to compensate for the total costs of the multistep biorefinery operations. Such pre-treatment processes require cost-efficient cooking chemicals. Due to its alkalinity and volatility, ammonia is a plausible option as a reusable chemical catalyst. In addition to the processes using anhydrous or low-moisture ammonia (Cayetano and Kim, 2017, 2018; Mittal et al., 2017; Flores-Gómez et al., 2018; Guo et al., 2018; Sakuragi et al., 2018; Zhou et al., 2018), aqueous ammonia pre-treatments have been studied intensively in recent years (Sipponen, 2015; Domanski et al., 2016; Phitsuwan et al., 2016; Chong et al., 2017; Li et al., 2017; Niemi et al., 2017; Tolbert et al., 2017; Yoo et al., 2017; Du et al., 2018; Huo et al., 2018; Zhu et al., 2018; An et al., 2019; Xiao et al., 2019). In contrast to anhydrous ammonia, pre-treatment of plant biomass with aqueous ammonia dissolves lignin that can be isolated from the spent cooking liquor. It is known that oxidative cleavage of lignin occurs when ammonolysis is performed under oxygen atmosphere at elevated temperatures (Lapierre et al., 1994; Nascimento et al., 1994; Capanema et al., 2006), but reports on the characterization and applications of ammonolysis lignin from actual biomass pre-treatments are scarce.

Some earlier works have investigated oxidative ammonolysis of technical lignins to produce slow-release nitrogen fertilizers (Nascimento et al., 1994; Ramírez et al., 1997; Capanema et al., 2001). Aqueous ammonia-based biomass pre-treatment processes have seen steady development because of the lower basicity and milder cooking temperatures compared to hydrothermal pre-treatments (Du et al., 2018; Huo et al., 2018; Zhu et al., 2019). However, one drawback of any alkaline pre-treatment is the consumption of effective alkali due to the alkaline hydrolysis of ester-linked moieties of biomass. A two-stage process with a pre-hydrolysis step could alleviate the neutralization issue. Such sequential pre-treatments involving aqueous ammonia have been studied recently (Chong et al., 2017; An et al., 2019; Xiao et al., 2019) but information is lacking regarding how the severity of the aqueous ammonia delignification affects the yield and applicability of soluble lignin along with the hydrolysability of the solid fraction.

In the present work, a two-stage hydrothermal-aqueous ammonia pre-treatment of wheat straw was studied. We used response surface modeling and fitted a combined severity parameter to evaluate the effects of various cooking conditions on the fractionation and enzymatic saccharification of wheat straw. The pre-treated straw and the lignin fractions were characterized using an array of techniques (elemental analysis, dye adsorption, ^31^P NMR and infrared spectroscopy, size-exclusion chromatography, and transmission electron microscopy) and the results are discussed in relation to the process conditions. Finally, using dye adsorption as a model system, we show that nitrogen-containing lignin holds potential as a broad-range adsorbent for cationic, anionic, and uncharged organic pollutants.

## Materials and Methods

### Materials

Commercial soda lignin (GreenValue SA, Switzerland) and soda lignin isolated from hydrothermally pre-treated wheat straw were used as reference materials in this study. Characterization of these lignins has been performed in the prior literature (Sipponen, 2015).

### Wheat Straw Pre-treatments and Isolation of N-lignin

Wheat straw (14 kg, dry basis) was collected from southern Finland during harvest, ground to pass a 1 mm screen in a Wiley mill, and subjected to hydrothermal (HT) pre-treatment at 140°C for 5 h (severity Log *R*_0_ = 3.8, see Equation 1). The solid fraction collected after water-washing of pre-treated straw was dried at 40°C to 10 wt-% moisture content and termed HT-straw. Small portions of HT-straw (12.5 g on dry basis) were subjected to aqueous ammonia extraction at a liquid to solid ratio of 12. The cubic experimental design varied non-isothermal target temperature (100, 130, 160°C) and ammonia concentration (0, 4, 12, 20%, w/w) in the aqueous phase. The actual temperature as a function of treatment time was recorded for the calculation of severity parameters. The solid fraction was washed with deionized water, dried under ambient conditions, and subjected to enzymatic hydrolysis.

**Scheme 1:**
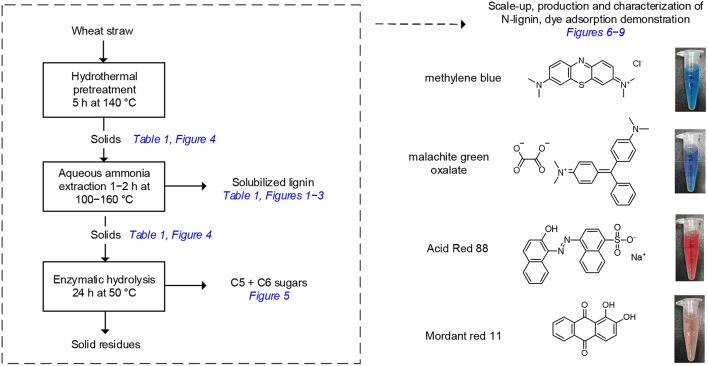
Overview of the research approach involving a two-stage pre-treatment and enzymatic hydrolysis of wheat straw, analysis of the liquid and solid fractions, scale-up and isolation of N-lignin for characterization and dye adsorption tests. Photographs show appearance of the dye solutions prior to adsorption.

A larger batch of lignin was isolated from wheat straw in a two-stage pre-treatment. In the first step, 33.4 kg of wheat straw at a liquid to solid ratio of 10 was subjected to HT pre-treatment at 177°C (Log *R*_0_ = 4.1). After separation of the liquid fraction, the solids were washed, pressed, and extracted with 7.5 wt% aqueous ammonia at a liquid to solid ratio of 7. Heating of the reactor to a maximum temperature of 140°C took ~3 h. The solid/liquid separation was made by filtration in a hermetic Nutsche equipment. Residual ammonia in the solid fraction was removed by evaporation at reduced pressure and captured in 6 M sulfuric acid. Lignin was precipitated from the spent aqueous ammonia cooking liquor (30 kg fraction sampled from the cooking liquor, pH 8.5) by acidification to pH 5 (100 mL of 6 M hydrochloric acid), filtration, four sequential washing steps by making a dilute water suspension and separating the liquid fraction from the lignin solids by centrifugation, and freeze-drying. The corresponding mass yield of the dry N-lignin from the initial HT-straw was 17%. The above described pre-treatments and resulting liquid and solid fractions obtained from wheat straw are summarized in Scheme 1.

### Combined Severity Parameter Fitting

Severity parameters have been previously used for acid- and base-catalyzed lignocellulose pre-treatments (Pedersen and Meyer, 2010). Here, we used a severity factor Equation (1) that combines the effects of time, temperature, and the alkaline catalyst on biomass during the aqueous ammonia pre-treatment.

(1) $$\begin{array}{l} Log\;M_{0}=Log\;R_{0}\;+\;Log\;C^{n} \end{array}$$

$R_{0}=\int_{0}^{t}\exp^{\frac{T\left(t\right)-100}{14.75}}dt$, where *T*(*t*) = treatment temperature at time *t*. The concentration of ammonia is given in the term *C*^*n*^ as weight percentage in the aqueous phase. The exponential factor *n* was obtained by iterative least squares linear fitting of the percentage of straw dissolved as a function of *Log M*_0_.

### Compositional Analysis of Lignin and Carbohydrates

The compositions of the straw fractions and N-lignin were determined following the two-stage sulfuric acid hydrolysis procedure (Sluiter et al., 2010). Sugar content of N-lignin was analyzed after hydrolysis of 20 mg dry material in 1.4 mL of 4 wt-% sulfuric acid (1 h, 121°C). The amount of dissolved lignin in the aqueous ammonia extracts was determined using the spectrophotometric method (Dence, 1992). Lignin concentration was calculated based on the absorbance reading at 280 nm using N-lignin for calibration (ε = 20.0 L/g/cm). Elemental carbon, hydrogen, nitrogen, and sulfur contents of various straw fractions and isolated ammonia lignin were analyzed with Perkin-Elmer (PE) 2400 Series II CHNS/O Analyzer. Sample weight was 2 mg.

### Characterization of Pre-treated Straw by Dye Adsorption

Adsorption of the cationic dye Azure B was used to estimate the accessible surface area of lignin (Sipponen, 2015). Adsorption of Azure B (0, 0.1, 0.3, 0.5, 0.7, 0.8, and 1.0 g/L) on 50 mg of HT-straw before and after aqueous ammonia extraction was performed at 25°C in 0.05 M Na-phosphate buffer (pH 7). The soluble dye concentration after 24 h contact time was calculated from the absorbance reading at 647 nm. The maximum monolayer adsorption capacity was obtained by non-linear fitting of the Langmuir equation to the adsorption isotherms. Additionally, adsorption capacities of all pre-treated straw fractions were determined at 0.1 g/L initial dye concentration to assess the effect of pre-treatment severity on the equilibrium adsorption capacity.

### Enzymatic Hydrolysis of Pre-treated Wheat Straw

Enzymatic hydrolysis assays were performed on the solid fractions either at 2 wt-% or 5 wt-% concentration of solids in the whole slurry with respective enzyme dosages of 15 FPU/g and 2 FPU/g. The enzyme mixture consisted on volume basis of 85% Econase CE (AB enzymes), 10% Novozyme 188 (Sigma-Aldrich/Novozymes), and 5% GC 140 (Genencor). The hydrolysis reactions were carried out in capped flasks agitated at 50°C. The liquid phase was sampled after 24 h reaction time for sugar analysis with HPLC. Briefly, the system included a Micro- Guard De-Ash pre-column (Bio-Rad, USA) connected to a SPO810 analytical column (Shodex). The analytical column was eluted with degassed deionized water at a flowrate of 0.7 mL/min isothermally at 60°C. A refractive index detector RID-10A was used to quantify monosaccharides identified based on their retention times and external calibration. Enzymatic hydrolysability was calculated as follows:

(2) $$\begin{array}{l} Enzymatic\;hydrolysability=\;100\%\cdot \frac{m_{sugar\;\left(enzymatic\;hydrolysis\right)}}{m_{sugar\;\left(acid\;hydrolysis\right)}} \end{array}$$

where *m*_*sugar* (*enzymatic hydrolysis*)_ and *m*_*sugar* (*acid hydrolysis*)_ are the total amounts of monosaccharides released from 1 g of pre-treated straw in enzymatic and analytical acid hydrolysis (two-step hydrolysis in concentrated and dilute sulfuric acid, see section Compositional Analysis of Lignin and Carbohydrates). Sugar recovery yield was calculated as follows:

(3) $$\begin{array}{l} Sugar\;recovery\;yield\;=\;\frac{m_{solid\;fraction}}{m_{HT-straw}}\cdot Enzymatic\;hydrolysability \end{array}$$

where *m*_*solid fraction*_ is the mass of straw recovered after aqueous ammonia extraction of *m*_*HT*−*straw*_ amount of HT-straw (both on dry basis). Reported results are mean values of two independent experiments.

### Characterization of Aqueous Ammonia-Soluble Lignins

Molecular weight distributions and weight average molar masses of lignin fractions from aqueous ammonia treatments were analyzed by aqueous high-performance size-exclusion chromatography (HPSEC). The first system was equipped with three Ultrahydrogel columns (Waters) eluted with aqueous 0.01 M sodium hydroxide containing 0.1 M sodium nitrate. The second system used a series of three PSS MCX 5 μm 300 mm × 8 mm, 100, 500, and 1,000 Å columns eluted with aqueous 0.1 M sodium hydroxide. Both systems were calibrated with poly(styrenesulfonate) standards and used a variable wavelength detector set at 280 nm.

N-lignin from the up-scaled process was analyzed by ^31^P NMR spectroscopy (Granata and Argyropoulos, 1995). The samples were phosphitylated using 2-chloro-4,4,5,5-tetramethyl-1,3,2-dioxaphospholane and analyzed as described more in detail elsewhere (Sipponen et al., 2017b) except that the relaxation delay d1 of 15 s was used in the present work. Reported results are mean values of two independent experiments.

FTIR spectra of ammonolysis lignin and wheat straw soda lignin (GreenValue SA, Switzerland) were recorded in the mid-infrared region (400–4,000 cm^−1^) at a resolution of 4 cm^−1^ by using a Bio-Rad FTS 6000 spectrometer (Digilab, Randolph, MA, USA) equipped with a MTEC PAC300 photoacoustic detector.

Transmission electron microscopy (TEM) images were recorded from drop-casted N-lignin on 3 nm carbon coated copper grids. FEI Tecnai 12 microscope was operated at 120 kV to acquire the images under bright field mode.

### Water Purification by Dye Adsorption

N-lignin and GreenValue wheat straw soda lignin were tested comparatively as adsorbents for water purification. Aqueous lignin dispersions (5 mL, 0.7 mg/mL) were adjusted to pH 6 and added to aqueous dye solutions of methylene blue, malachite green oxalate, acid red 88 (5 mL, 0.2 mM), or mordant red 11 (5 mL, saturated solution) and magnetically stirred at 22°C during 8 h along with dye solutions at similar initial concentrations as above but without lignin. Absorbance spectra of the filtered (0.45 μm pore size to ensure exclusion of any submicrometer lignin particles that may have affected spectrophotometric measurements) liquid phases were recorded at 200–800 nm. Decolourization was calculated as percentage reduction of absorbance (at λ_max_ in the visible region) compared to the absorbance of the dye solutions without adsorbents. Reported results are mean values of two independent experiments.

## Results and Discussion

The objective of this work was to develop a two-stage hydrothermal-aqueous ammonia pre-treatment of wheat straw with subsequent enzymatic saccharification and valorization of the isolated lignin fraction. The pre-treatment process was scaled up to allow for production of N-lignin for dye adsorption tests. It is well-known that efficient saccharification of the pre-treated biomass requires opening up the recalcitrant lignocellulose structure by the removal or modification of lignin. Our first important task was therefore to investigate the mass balances and compositional changes as a result of the pre-treatment process.

### Fractionation of Wheat Straw in the Two-Stage Pre-treatment

The effects of ammonia concentration, treatment time and temperature on ammonolysis of technical lignins have been studied in a series of detailed investigations (Capanema et al., 2001, 2002, 2006). Here, our objective was to use aqueous ammonia to fractionate hydrothermally pre-treated wheat straw (HT-straw) for enzymatic saccharification and isolation of N-lignin for valorization. First, we used response surface modeling to assess how extraction temperature and ammonia concentration influenced dissolution of HT-straw in aqueous ammonia. The yields of soluble lignin and solid fractions are given in Table 1 along with the carbohydrate and lignin compositions of the solids. In general, dissolution of HT-straw increased as the ammonia concentration and treatment temperature increased. The lowest yield of the solid fraction was 68.6%, while the soluble lignin fraction was obtained at a maximum yield of 15.8% following the treatment at 160°C in 20% aqueous ammonia. The resulting solid straw fraction contained 13.2% of lignin, compared to 23.6% lignin content in HT-straw. It is noteworthy that almost as good yield of soluble lignin (14.2%, entry 10 in Table 1) was obtained with 12 wt-% of ammonia at 160°C. This observation suggests that the extent of lignin removal from HT-straw could be increased by increasing the treatment temperature. However, previous results indicate that maintaining hemicellulose and a low content of residual lignin can be beneficial to the enzymatic hydrolysability, presumably by avoiding the collapse of the porous cell wall matrix (Pihlajaniemi et al., 2016; Zhang et al., 2016; An et al., 2019) While the content of pentose sugars decreased slightly, extraction of lignin enriched the solid fractions with cellulose and hemicellulose, reaching 78.1% total carbohydrate content at the highest treatment temperature and ammonia concentration. Calculated from the data in Table 1, the mass balance closure decreased only slightly from 94 to 84% in response to the increasing treatment severity. Two aqueous ammonia pre-treatments that were carried out under identical conditions (130°C, 12 wt% ammonia solution) gave similar results, indicating that the reproducibility of the experiments was sufficient for the modeling of the data.

**Table 1:** Conditions of aqueous ammonia extraction, mass yield of solids and soluble lignin, and composition of the resulting solid fractions before enzymatic saccharification.

**Entry**	**Pre-treatment**		**Mass yield**		**Composition of solids, % dry weight basis**			
	NH_3_ (wt-%)	T (^°^C)	Solids (%)	Soluble lignin (% of HT-straw)	Glc*^a^*	Xyl*^a^*	Ara*^a^*	Lignin*^b^*
1	0	0	100.0	0	39.8 ± 0.2	23.5 ± 0.4	2.7 ± 0.0	23.6 ± 0.2
2	0	140	80.2	n.a.	41.5 ± 0.3	21.2 ± 0.3	1.8 ± 0.1	23.9 ± 0.0
3	0	100	92.7	1.3	43.6 ± 0.5	21.1 ± 1.2	0.8 ± 0.1	22.9 ± 0.2
4	4	100	84.9	6.5	47.0 ± 0.6	20.9 ± 1.2	0.8 ± 0.2	22.0 ± 0.0
5	4	130	80.8	9.4	51.1 ± 0.3	20.0 ± 0.5	1.9 ± 1.0	19.7 ± 0.5
6	4	160	77.1	11.6	54.5 ± 0.7	18.3 ± 0.5	0.7 ± 0.2	19.5 ± 0.3
7	12	100	83.8	6.6	48.7 ± 0.5	20.5 ± 0.3	0.5 ± 0.5	20.6 ± 0.5
8	12	130	75.4	12.0	53.5 ± 1.3	18.6 ± 1.2	0.5 ± 0.5	17.3 ± 0.4
9	12	130	76.3	10.7	52.4 ± 0.4	19.2 ± 1.0	0.4 ± 0.4	18.0 ± 0.5
10	12	160	72.7	14.2	54.8 ± 0.8	18.1 ± 1.0	0.5 ± 0.2	16.2 ± 0.8
11	20	100	80.2	8.3	51.7 ± 0.6	20.9 ± 0.1	0.7 ± 0.2	20.1 ± 0.7
12	20	130	73.2	11.5	54.8 ± 0.7	17.5 ± 0.8	0.4 ± 0.4	16.2 ± 0.7
13	20	160	68.6	15.8	59.2 ± 0.5	18.2 ± 0.5	0.7 ± 0.2	13.2 ± 0.2

a *Calculated as anhydrous sugars*;

b *Includes acid-insoluble and acid-soluble lignin. n.a., not analyzed. Entries: 1 = original wheat straw, 2 = wheat straw subjected to 5 h hydrothermal (HT) treatment at 140°C; 3 = 100°C water-extracted HT-straw (entry 2), 4–13 = aqueous ammonia pre-treated HT-straw (entry 2)*.

**Figure 1:**
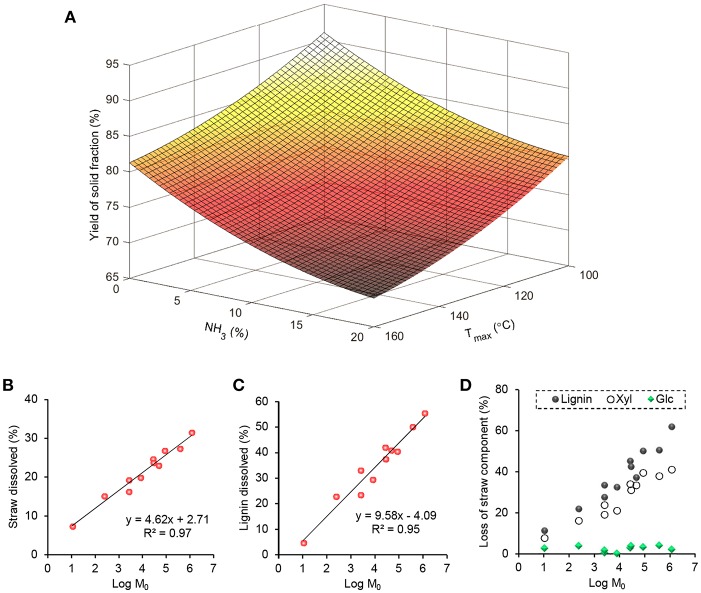
Optimization and modeling of aqueous ammonia extraction of hydrothermally pre-treated wheat straw (HT-straw). **(A)** Response surface showing the effects of aqueous ammonia and maximum treatment temperature on the dry weight yield of solid fractions. Linear fitting of percentage dissolution of **(B)** straw (% of HT-straw dry weight) and **(C)** lignin (% of lignin dry weight of HT-straw) as a function of the combined severity parameter: $Log\;M_{0}=Log\;R_{0}\;+\;Log\;C^{2.23}$. **(D)** Loss of glucose, xylose (both anhydrous basis) and total lignin from straw, based on mass balance calculated from the data in Table 1.

The response surface shown in Figure 1A was obtained when the yield of solid straw fraction from the pre-treatment was fitted to a quadratic model (*R*^2^ = 0.97). Though such three-dimensional plots are effective for the visualization of data, combination of the pre-treatment parameters (ammonia concentration, treatment time, and temperature) into a single parameter allows for further analysis of enzymatic hydrolysability of the solid fractions from the pre-treatments at various severities. The best-fit of ammonia concentration and treatment temperature produced a coefficient of determination (*R*^2^) of 0.97 and an exponential factor (*n*) of 2.23 for the combined severity parameter Log M_0_ (Figure 1B). A higher *n*-value of 3.90 has been fitted for lignin removal from cotton stalks by sodium hydroxide treatment (Silverstein et al., 2007), and also used to calculate pre-treatment severity of aqueous ammonia soaking (Huo et al., 2018). However, we reasoned that differences in biomass type (in our case hydrothermally pre-treated straw) and basicity (lower with ammonium hydroxide than sodium hydroxide) rationalize using a different value here. Effectively, the extent of dissolution of lignin increased from 5 to 55% in linear correlation (*R*^2^ = 0.95) with the severity parameter, validating its usefulness in comparison of the pre-treatment conditions (Figure 1C). Taking into account the yield of the solid fraction, we calculated the loss of individual straw components from the mass balances (Figure 1D). There was a negligible loss of glucose (≤4%), but the loss of xylose (maximum 41%) correlated (linear regression, *R*^2^ = 0.94) with the loss of lignin (maximum 62%). This simultaneous removal of lignin with xylose suggest that the two components are closely associated with each other. To shed light into the interactions of these polymers, we further analyzed the spent cooking liquor fractions from the ammonia treatments.

### Characterization of Aqueous Ammonia-Soluble Lignin

**Figure 2:**
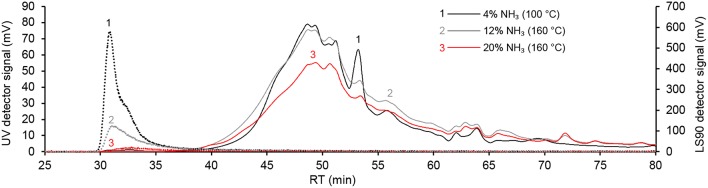
Aqueous SEC traces of lignins extracted from hydrothermally pre-treated wheat straw with aqueous ammonia under varied severity. Dashed lines: light scattering detector (90°) signal.

Aqueous size-exclusion chromatography was used to analyze molecular weight distribution of the lignins solubilized during the aqueous ammonia treatment. Figure 2 shows three chromatograms recorded from lignins obtained at low (*Log M*_0_ = 2.4), medium (*Log M*_0_ = 5.6), and high (*Log M*_0_ = 6.1) severities. The most obvious observation that can be made from the chromatograms is the disappearance of the sharp static light scattering detector signal at 30.8 min as the severity increased. This peak outside of the column calibration range was likely caused by lignin-carbohydrate complexes (LCCs) that contained relatively few lignin fragments, since the associated UV detector signal was very low. Although further analysis of LCCs was out of scope of the present work, it is noted that LCCs from wood and grass biomass have been characterized before (Watanabe et al., 1989; Lawoko et al., 2006; Yuan et al., 2011). The most pre-dominant covalent linkages in LCCs are phenyl glycoside, γ-ester, and benzyl ether (Yuan et al., 2011). We expect that cleavage of these LCCs occurred under the harsh alkaline conditions of the aqueous ammonia pulping enabled retaining a majority of the hemicelluloses in the solid straw fraction. In turn, there was a weak correlation of increasing weight average molecular weight (${\bar{M}}_{w}$) of aqueous ammonia-solubilized lignin fractions with increasing severity (*R*^2^ = 0.48) or percentage dissolution of lignin (*R*^2^ = 0.58). The ${\bar{M}}_{w}$ of the 10 lignin fractions varied from 2,420 g/mol to 3,880 g/mol, which are in the similar range compared to the values of aqueous ammonia lignins isolated from wheat straw (2,200 g/mol) and *Miscanthus* × *giganteus* (3,140 g/mol) (Kondo et al., 1992; Vanderghem et al., 2011).

**Figure 3:**
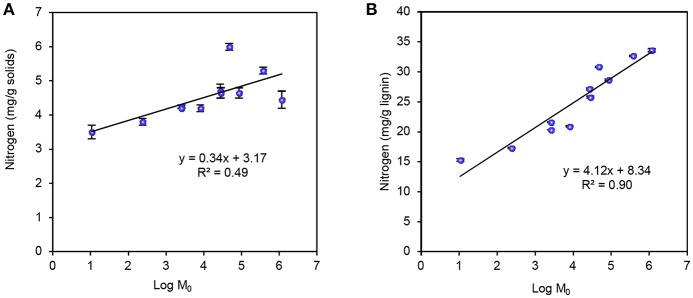
Effect of severity of aqueous ammonia pre-treatment (*Log M*_0_) on nitrogen content of recovered solid fractions **(A)** Nitrogen content of the pre-treated solid fractions. **(B)** Nitrogen content calculated relative to the lignin content of the solid fractions.

Aqueous ammonia was expected to cause incorporation of nitrogen by ammonolysis of lignin in HT-straw. Indeed, elemental analysis revealed that nitrogen content of the partially delignified solid fractions increased as the pre-treatment severity increased (Figure 3A). A stronger linear correlation to the severity parameter was observed when the nitrogen content was related to the lignin content of the pre-treated straw fractions (Figure 3B). Therefore, nitrogen seemed to bind mainly to lignin as suggested by earlier literature (Lapierre et al., 1994; Potthast et al., 1996).

### Adsorption of Cationic Dye on Pre-treated Straw With Different Lignin Content

**Figure 4:**
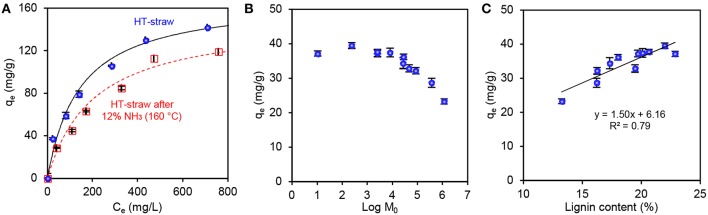
Characterization of aqueous ammonia extracted HT-straw by cationic dye adsorption. **(A)** Langmuir model fitted to adsorption isotherm of Azure B on HT-straw and HT-straw pre-treated with 12% aqueous ammonia at 160°C. Equilibrium adsorption capacities (0.1 g/L initial dye concentration) as a function of **(B)** The severity parameter (Log M_0_) of aqueous ammonia extraction. **(C)** Lignin content of pre-treated straw. Data points show mean values ± one standard deviation.

The compositional and chemical analyses discussed above proved that aqueous ammonia treatment caused severity-dependent lignin removal from HT-straw. Adsorption of the cationic dye Azure B on pre-treated solid fractions was used to assess whether these modifications caused changes in their accessible surface areas. A further aim was to assess how the accessible surface area of the pre-treated straw solids relates to their enzymatic hydrolysability. The adsorption isotherms of Azure B on the HT-straw before and after the aqueous ammonia pre-treatment are shown in Figure 4A. The aqueous ammonia pre-treatment caused a clear drop in the adsorption capacity due to the removal of lignin from the solid straw fractions (Table 1). This was expected on the basis of prior studies that have shown that lignin has a significantly higher adsorption capacity for Azure B compared to that of cellulose (Sipponen, 2015).

A fixed initial dye concentration (0.1 g/L) was used to compare adsorption capacities and thus evaluate accessible surface area of lignin in relation to the pre-treatment severity. The equilibrium adsorption capacity remained unchanged at low severity levels, but eventually decreased from 188 to 124 mg/g when the severity increased from 2.4 to 6.1 (Figure 4B). This decrease in the adsorption capacity correlated (*R*^2^ = 0.79, *p* < 0.01, Figure 4C) with the decreasing lignin content of the solid fractions, suggesting that aqueous ammonia removed lignin solvolytically in contrast to melting and degradation of lignin under acidic and hydrothermal conditions (Selig et al., 2007).

### Enzymatic Hydrolysability of Solids After Aqueous Ammonia Extraction

**Figure 5:**
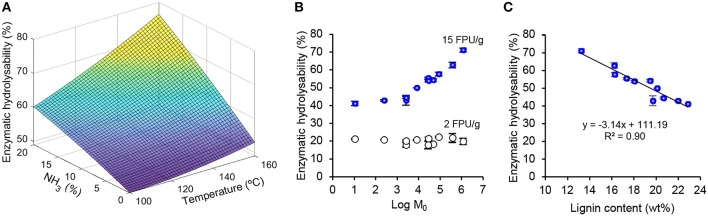
Enzymatic hydrolysability (24 h reaction time) of pre-treated straw solid fractions. **(A)** Response surface plot showing effects of ammonia concentration and treatment temperature on enzymatic hydrolysability (15 FPU/g). **(B)** Hydrolysabilities at 15 FPU/g (filled circles) and 2 FPU/g (open circles) activity dosages as a function of pre-treatment severity *Log M*_0_; **(C)** Hydrolysability (15 FPU/g) as a function of lignin content of pre-treated straw prior to enzymatic hydrolysis. Data points show mean values ± one standard deviation.

High yield and volumetric productivity of monomeric sugars from pre-treated biomass is central for the production of bioethanol or other products via microbial conversion routes (Pihlajaniemi et al., 2014). The solid fractions were collected after the various pre-treatments and subjected to enzymatic hydrolysis at low (2 FPU/g) and high (15 FPU/g) cellulase enzyme activity dosages. A response surface plot was created by fitting the enzymatic hydrolysabilities to a quadratic model (Figure 5A, *R*^2^ = 0.95). The response surface indicates that the enzymatic hydrolysability increased with increasing ammonia concentration and treatment temperature. The shape of the surface suggests that additional increment in hydrolysability could have been reached by further increasing severity of the aqueous ammonia pre-treatment. Similar conclusion can be reached by correlating enzymatic hydrolysability to the combined pre-treatment severity (Log M_0_) in Figure 5B. There was a clear trend of increasing hydrolysability as the pre-treatment severity increased when using 15 FPU/g enzyme dosage. Enzymatic hydrolysability remained at 41–43% until the pre-treatment severity value of 3.4, but increased linearly thereafter to reach a value of 71.1% after the 24 h reaction. Taking into account the mass balances, the most severe pre-treatment conditions (20% NH_3_, 160°C, Log M_0_ = 6.1) led to the highest sugar recovery yield from HT-straw (39.8%), representing a clear augmentation compared to 27.8% obtained from HT-straw without aqueous ammonia treatment.

Inverse linear correlation of enzymatic hydrolysability with the lignin content of the pre-treated solids (R^2^ = 0.90, Figure 5C) also points to the fact that further selective removal of lignin could increase the sugar recovery yield in the overall process. Moreover, the enzymatic hydrolysability (15 FPU/g) showed an inverse correlation with the equilibrium Azure B adsorption capacity (0.1 g/L initial dye concentration, *R*^2^ = 0.89). That essentially similar *R*^2^ values were obtained from the two factors is opposite to what has been reported following the autohydrolysis pre-treatment that melts lignin and reduces lignin surface area without major changes in the lignin content (Sipponen et al., 2014). In fact, multiple physical and chemicals changes occur in plant biomass during thermochemical pre-treatment (Pedersen and Meyer, 2010; Pihlajaniemi et al., 2016; Paës et al., 2017). Although the single-parameter correlations do not necessarily indicate causation, the results presented here support the earlier conclusion that cationic dye adsorption can be used as an indirect indicator of the constraint from residual lignin to enzymatic hydrolysis of polysaccharides (Sipponen et al., 2014).

**Figure 6:**
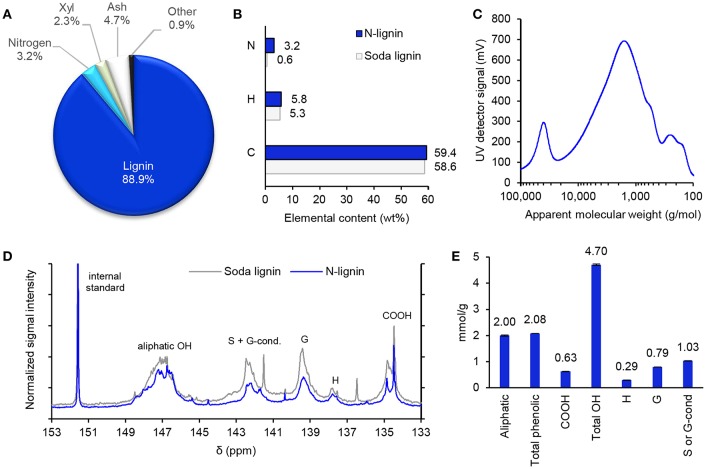
**(A)** Composition of N-lignin. **(B)** Carbon, hydrogen, and nitrogen contents of N-lignin compared to the published values of wheat straw soda lignin extracted from HT-straw (Sipponen, 2015). **(C)** HPSEC trace of N-lignin. **(D)** ^31^P NMR spectra of soda lignin (Sipponen, 2015) and N-lignin. **(E)** Quantities of aliphatic and phenolic hydroxyl groups and carboxylic acid groups of N-lignin based on ^31^P NMR analysis. Error bars indicate ± one standard deviation relative to the mean value.

Several wood and grass biomass types have been subjected to aqueous ammonia pre-treatment prior to the enzymatic hydrolysis step for sugar production (Du et al., 2018; Huo et al., 2018; An et al., 2019). The combination of ammonia pulping with ultrasound treatment (Du et al., 2018) and oxidative agents such as hydrogen peroxide (Huo et al., 2018) or oxygen gas (An et al., 2019) has increased subsequent enzymatic hydrolysability at least in a few cases. Sequential pre-treatment with dilute acid and aqueous ammonia has been found beneficial regarding hydrolysability of cellulose (An et al., 2019). Recent studies reported a high sugar recovery yield of 78% (Du et al., 2018) and an enzymatic digestibility of 84%, (Huo et al., 2018) but these were achieved with 30 FPU/g (glucan) cellulase dosage, i.e., much higher dosages than the ones used here. In addition to differences in feedstock types and pre-treatment conditions, it is also important to note that we obtained distinctively different results when a low cellulase activity was used in the saccharification reaction (Figure 6A). In contrast to what was observed at 15 FPU/g, the hydrolysability at 2 FPU/g remained low (~20%) and essentially unchanged regardless of the severity. In contrast to the present work, An et al. (2019) achieved a reasonably high (72%) glucan conversion at 3 FPU/g cellulase dosage, which may be due to the oxygen pressure that enhanced lignin removal in the ammonolysis of dilute acid pre-treated corn stover. Our results suggest that nitrogen incorporation in the residual lignin is an important factor impeding enzymatic hydrolysis at the low cellulase dosage and regardless of the high extent of delignification. One plausible explanation is the competitive adsorption of cellulases on nitrogen-containing residual lignin. This reasoning is in agreement with our results (Figure 3B) and recent findings showing a significantly higher affinity and binding strength of cellulase on ammonia lignin residue than on organosolv lignin (Yoo et al., 2017).

### Characterization of N-lignin From the Up-Scaled Process

To get a more comprehensive understanding of the nitrogen-containing lignin and to isolate a sufficient amount of material for application tests, a larger batch of hydrothermally pre-treated wheat straw was extracted with aqueous ammonia, and the isolated N-lignin was characterized. The fractionation process involved hydrothermal treatment at 177°C followed by aqueous ammonia extraction (7.5% NH_3_, 140°C). These conditions were selected to remove a majority of hemicelluloses before the aqueous ammonia extraction step (20% solubilization of wheat straw, entry 2 in Table 1). Compositional analysis showed that N-lignin mainly contained (on dry weight basis) lignin (88.9 ± 0.6%), xylose (2.3 ± 0.3%, anhydrous basis), ash (4.7 ± 0.1%), and trace amounts of other components (Figure 6A). The lignin fraction was thus quite pure and had a low amount of sugar contaminants. Despite their similar carbon and hydrogen contents, the nitrogen content of N-lignin (3.2%) was ~5 times as high as that of soda lignin that was previously isolated from the same batch of HT-straw (Figure 6B).

The mass recovery yield of N-lignin (approximately 5% of hydrothermally pre-treated straw dry weight) was lower than expected (12%) based on the severity value (*Log M*_0_ = 4.8), but its nitrogen content (32 mg/g) was similar as those of the residual lignins in pre-treated straw at comparable severities (Figure 3B). The lower extent of lignin dissolution is likely due to the higher temperature (177°C) used in the hydrothermal treatment compared to the milder temperature in the small scale experiments (140°C). In fact, it is well known that acid-catalyzed formation of condensed lignin under high temperatures hampers extraction of lignin from lignocellulose (Lora and Wayman, 1978). Alkaline pulping involves fragmentation of the side chain region of lignin and generation of free phenolic hydroxyl groups (Sipponen et al., 2017a). Molecular weight is one of the key parameters of polymers, and hence we recorded the molecular weight distribution of N-lignin using SEC (Figure 6C). Most of the molecules eluted at apparent molecular weights between 400 g/mol and 20,000 g/mol, with ${\bar{M}}_{w}$of 3,100 g/mol (PDI = 2.3) that was in the same range as observed above in the small scale experiments.

Quantitative ^31^P NMR spectra were recorded from N-lignin to get further insight of the chemical changes that may have occurred during the extraction process. The spectrum shown in Figure 6D differed slightly from the one recorded earlier from the wheat straw soda lignin (Sipponen, 2015). Quantification of the hydroxyl group moieties (on dry weight basis) revealed that N-lignin contained 4% more aliphatic hydroxyls, while carboxylic acid and free phenolic hydroxyl groups were 24% and 9% lower compared to the values for the soda lignin (Figure 6E). These small differences may result from the stronger alkalinity of sodium hydroxide compared to ammonium hydroxide, and effectively a lower extent of oxidation with the latter. Yoo et al. (2017) recently reported that treatment of poplar lignin with 5% aqueous ammonia at 180°C caused a moderate increase in the content of C5-substituted phenolic hydroxyl groups with a concomitant loss of G and H type of phenolic hydroxyl and aliphatic hydroxyl groups.

**Figure 7:**
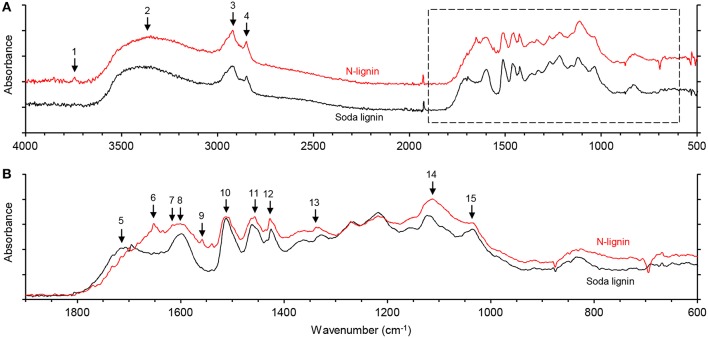
FT-IR spectra of N-lignin and GreenValue wheat straw soda lignin. **(A)** Full spectra. **(B)** Overlaid spectra at 1900–600 cm^−1^. See text and Table 2 for signal assignments.

FTIR spectroscopy was used to analyze the chemical structure of N-lignin in comparison to commercial wheat straw soda lignin. Figure 7 shows typical lignin signals in both of the lignins that were assigned according to Faix (1992). The signal intensities were calculated relative to the baseline values at 1,900 cm^−1^. The two lignins showed similar intensities at 1,512 cm^−1^ (aromatic skeletal vibrations) but differences were detected in bands indicating the presence of amides at 3,744 cm^−1^, 1,653 cm^−1^, 1,558 cm^−1^, and 1,427 cm^−1^ (Fu et al., 1994; De Campos Vidal and Mello, 2011; Yoo et al., 2014). Relative signal intensities of these amide bands were stronger in N-lignin than in soda lignin (Table 2). These results indicate that the formation of amides occurs under considerably milder ammonolysis conditions than those used in the previous studies (Lapierre et al., 1994; Potthast et al., 1996).

**Table 2:** Signal intensities relative to the baseline value at 1,900 cm^−1^ of N-lignin and GreenValue wheat straw soda lignin, and differences between the two.

**No**	**cm^**−1**^**	**N-lignin**	**Soda lignin**	**Difference**	**Assignment**
1	3744	1.30	1.28	0.02	Amide N-H stretching
2	3388	3.02	2.77	0.13	O–H stretching
3	2920	3.32	2.89	0.43	C–H stretch, methyl, and methylene groups
4	2849	2.84	2.44	0.40	C–H stretching, methyl, and methylene groups
5	1717	2.07	2.33	−0.26	C = O stretching, unconjugated carbonyl
6	1653	3.02	2.19	0.82	Amide I (C = O stretching)
7	1636	2.58	1.78	0.80	Imine (Shiff base) C = N stretching
8	1601	3.01	2.73	0.28	Ar. skeletal vibration; S>G
9	1558	2.58	1.78	0.80	Amide II (C–N stretch coupled with N–H bending)
10	1512	3.21	3.18	0.04	Ar. skeletal vibration; G>S
11	1456	3.21	2.92	0.29	C–H deform. methyl and methylene groups
12	1427	3.16	2.78	0.38	Ar. skeletal vibrations and C–H in-plane deform.
13	1339	2.92	2.60	0.31	Amide III (N–H in-plane bending coupled with C–N stretching plus C–H and N–H deformation); S ring plus G-cond
14	1113	3.71	3.20	0.51	Ar. C–H in-plane deform.; sec. alcohol; C = O stretching
15	1036	3.04	2.87	0.17	Ar. in-plane C-H deform.; C-O deform.; C = O stretching (unconj.)

The signal intensity of the unconjugated carbonyl band at 1,717 cm^−1^ was slightly lower in N-lignin compared to soda lignin. In accordance with our results, Yoo et al. (2017) reported that aqueous ammonia treatment of cellulolytic enzyme lignin (CEL) resulted in lower abundance of unconjugated carbonyl moieties. We postulate that the drop in unconjugated carbonyl moieties was due to the Schiff base formation during the aqueous ammonia treatment. This speculation is supported by the appearance in N-lignin of a new band at 1,636 cm^−1^ that was assigned to the C = N stretching in imine (Dos Santos et al., 2005), and though overlapped with other signals in this region, the band was absent in soda lignin.

**Figure 8:**
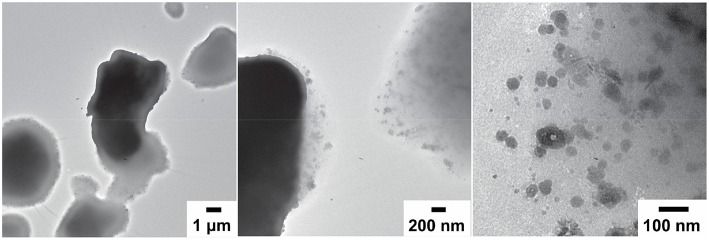
TEM micrographs of N-lignin dried from aqueous suspension.

TEM imaging was lastly employed to get a better understanding of the nanoscaled morphology of the N-lignin. It showed micrometer-sized flakes that contained partially aggregated nanoparticles (Figure 8). The smallest particles were ~20 nm and the majority of them were <100 nm in diameter. We anticipated that this fine morphology combined with the incorporated nitrogen could be beneficial for adsorption and flocculation in water remediation applications. Therefore, we carried out proof-of-concept experiments on dye adsorption from aqueous media.

### Water Purification Using the N-lignin Particles

**Figure 9:**
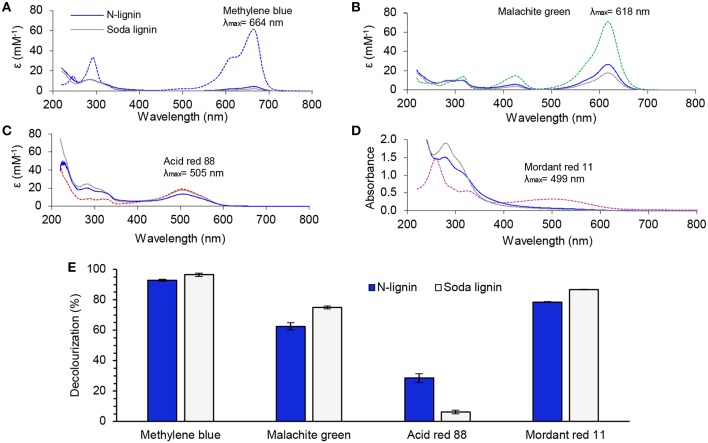
Dye adsorption (8 h, pH 6, 22°C) on lignins. UV-Vis absorbance spectra showing apparent dilution-corrected molar absorptivity of aqueous dye solutions before (dashed spectra) and after adsorption on N-lignin (blue spectra) and GreenValue wheat straw soda lignin (gray spectra): **(A)** Methylene blue; **(B)** Malachite green oxalate; **(C)** Acid red 88; **(D)** Absorbance spectra of Mordant red 11 before and after adsorption. **(E)** Decolourization (absorbance decrement) of the dye solutions by the different lignins. Error bars indicate ± one standard deviation relative to the mean value.

Due to their negative charge, many lignins have been shown to adsorb cationic dyes such as methylene blue (Yu et al., 2016; Albadarin et al., 2017) and malachite green (Tang et al., 2016) but this is of limited use in practical applications requiring purification of complex wastewaters. Hence our hypothesis was that the N-lignin could be more universal adsorbent due to its zwitterion character and hence we tested also anionic and neutral dyes. N-lignin and wheat straw soda lignin (GreenValue) were compared as adsorbents for two cationic dyes (methylene blue and malachite green), one anionic dye (acid red 88), and one uncharged dye (mordant red 11). The concentration of cationic and anionic dyes in the adsorption experiments was 0.1 mM. Due to its low water-solubility, a filtered saturated solution of mordant red 11 was used. Absorbance spectra were recorded after 8 h contact time with the adsorbents, and decolourization was calculated from the absorbance maxima in the visible wavelength region (Figures 9A–D).

Both of the lignins turned out to be effective adsorbents for methylene blue, with 93–96% decolourization and 84–86 mg/g (non-maximum) equilibrium adsorption capacities, respectively, for N-lignin and soda lignin (Figure 9E). In the case of malachite green the highest extent of 75% decolourization was obtained with soda lignin, compared to 63% with N-lignin. As mentioned above, anionic dyes are more demanding pollutants to remove by adsorption due to their electrostatic repulsion with negatively charged lignins. It was thus important to observe that N-lignin was clearly more effective adsorbent for Acid red 88 (29% decolourization) than soda lignin (6% decolourization). In the case of the uncharged dye mordant red 11, 87% decolourization was reached with soda lignin, a slightly higher value compared to 79% with N-lignin. This minor difference could be related to differences in surface area or adsorption sites of the two lignins, but further analysis of this matter was beyond the scope of the present work.

One important feature of any adsorbent is its structural stability in the wastewater. Due to their pH-dependent solubility, leaching of lignin from solid particles is common in aqueous media. Interestingly, due to the electrostatic complex formation absorbance reading at 280 nm did not indicate obvious leaching of lignins in the presence of cationic dyes, unlike in the presence of anionic and non-charged dyes (Figures 9A–D). Hydrogels represent another means to stabilize lignin-based adsorbents. Yu et al. (2016) synthesized hydrogels by grafting acrylic acid on the lignosulfonate backbone, and reported that the maximum equilibrium adsorption capacity of methylene blue reached 2,013 mg/g. The hydrogel was quite stable, since the adsorption capacity decreased only to 1,681 mg/g after four desorption/reuse cycles. Combination of lignin with chitin (Duan et al., 2018) and chitosan (Albadarin et al., 2017) has been made to form structurally stabilized composites for water purification. Tang et al. (2016) grafted acrylamide to lignosulfonate, and cross-linked the aromatic rings with formaldehyde under acidic conditions. Others have synthesized cationic lignin for adsorption and flocculation of pollutants (Wahlström et al., 2017). These promising results encourage to continue water remediation research with chemically tailored lignins and colloidal lignin particles (Sipponen et al., 2017b). In retrospect, the sorption of not only cationic but also anionic and uncharged dyes on N-lignin may explain why cellulases tend to adsorb on residual lignin in ammonia pre-treated straw. Cellulases contain charged and uncharged amino acids that may bind non-covalently with amphiphilic N-lignin, which may explain the adsorption affinity of cellulases on ammonia-treated lignin (Yoo et al., 2017).

## Conclusions

This work had two interlinked objectives. The first was to optimize aqueous ammonia extraction of hydrothermally pre-treated wheat straw to isolate lignin and facilitate enzymatic hydrolysis of the cellulosic fraction. The second important goal was to provide improved understanding of the isolated nitrogen-containing lignin, and to assess its valorization as adsorbent in water remediation. Aqueous ammonia extraction improved accessibility to hydrolytic enzymes of the straw polysaccharides mainly by removing lignin without extensive oxidation. However, incorporation of nitrogen in wheat straw lignin reduced hydrolysability at low enzyme activity dosages, presumably by competitive adsorption of cellulases. The nitrogen-containing lignin showed beneficial broad spectrum adsorption of anionic, cationic, and uncharged organic dyes from aqueous solutions. Besides the demonstrated water purification applications, our results suggest that N-lignins could find use as adsorbents in enzyme immobilization.

## Data Availability

All datasets generated for this study are included in the manuscript. Raw data is available from the authors upon a reasonable request.

## Author Contributions

MS carried out the experimental work and analyzed the results with input from MÖ. MS wrote the manuscript with input and critical comments from MÖ.

### Conflict of Interest Statement

The authors declare that the research was conducted in the absence of any commercial or financial relationships that could be construed as a potential conflict of interest.
